# Control of Avian Influenza in Poultry

**DOI:** 10.3201/eid1209.060430

**Published:** 2006-09

**Authors:** Ilaria Capua, Stefano Marangon

**Affiliations:** * Istituto Zooprofilattico Sperimentale delle Venezie, Legnaro, Padova, Italy

**Keywords:** avian influenza, vaccination, control, monitoring, biosecurity, DIVA, synopsis

## Abstract

Avian influenza, listed by the World Organization for Animal Health (OIE), has become a disease of great importance for animal and human health. Several aspects of the disease lack scientific information, which has hampered the management of some recent crises. Millions of animals have died, and concern is growing over the loss of human lives and management of the pandemic potential. On the basis of data generated in recent outbreaks and in light of new OIE regulations and maintenance of animal welfare, we review the available control methods for avian influenza infections in poultry, from stamping out to prevention through emergency and prophylactic vaccination.

## Abstract 2

Vaccines that enable detection of field exposure to any AI would be ideal.

Avian influenza (AI), which emerged from the animal reservoir, represents one of the greatest recent concerns for public health. Compared with the number reported for the past 40 years, the number of outbreaks of AI in poultry has increased sharply during the past 5 years. The number of birds involved in AI outbreaks has increased 100-fold, from 23 million from 1959 through 1998 to >200 million from 1999 through 2005 (*1*). Since the late 1990s, AI infections have assumed a completely different profile in the veterinary and medical scientific communities. Some recent outbreaks have been minor, but other epidemics, such as the Italian 1999–2000, the Dutch 2003, the Canadian 2004, and the ongoing Eurasian, have been more serious. They have led to devastating consequences for the poultry industry, negative repercussions on public opinion, and, in some instances, created major human health issues, including the risk of generating a new pandemic virus for humans through an avian-human link.

Influenza viruses are segmented, negative-strand RNA viruses that are placed in the family *Orthomyxoviridae* in 3 genera: *Influenzavirus A*, *B*, and *C*. Influenza A viruses are the only type reported to cause natural infections of birds and are further divided into subtypes according to antigenic characteristics of the surface glycoproteins hemagglutinin (H) and neuraminidase (N). At present, 16 hemagglutinin subtypes (H1–H16) and 9 neuraminidase subtypes (N1–N9) have been identified. Each virus has one H and one N antigen, apparently in any combination; all subtypes and most possible combinations have been isolated from avian species.

Influenza A viruses that infect poultry can be divided into 2 distinct groups according to the severity of disease they cause. The most virulent viruses cause highly pathogenic avian influenza (HPAI), a systemic infection in which death rates for some susceptible species may be as high as 100%. These viruses have thus far been restricted to strains that belong to the H5 and H7 subtypes and have a multibasic cleavage site in the precursor of the hemagglutinin molecule. HPAI is a lethal infection in certain domestic birds (e.g., chickens and turkeys) and has a variable clinical effect (may or may not cause clinical signs and death) in domestic waterfowl and wild birds. The potential role of wild birds and waterfowl as reservoirs of infection by HPAI strains has been described for only the Asian HPAI virus H5N1. The ecologic and epidemiologic implications of this unprecedented situation are not predictable.

On the contrary, viruses that belong to all subtypes (H1–H16) that lack the multibasic cleavage site are perpetuated in nature in wild bird populations. Feral birds, particularly waterfowl, are the natural hosts for these viruses and are therefore considered an ever-present source of viruses. Since their introduction into domestic bird populations, these viruses have caused low-pathogenicity avian influenza (LPAI), a localized infection that results in mild disease, primarily respiratory disease, depression, and egg-production problems. Theories suggest that HPAI viruses emerge from H5 and H7 LPAI progenitors by mutation or recombination (*2**,**3*), although >1 mechanism is likely. This theory is supported by findings from phylogenetic studies of H7 subtype viruses, which indicate that HPAI viruses do not constitute a separate phylogenetic lineage or lineages but appear to arise from nonpathogenic strains (*4**,**5*); this indication is supported by the in vitro selection of mutants virulent for chickens from an avirulent H7 virus (*6*).

Such mutation probably occurs after the viruses have moved from their natural wild-bird host to poultry. However, the mutation to virulence is unpredictable and may occur very soon after the virus is introduced to poultry or after the LPAI virus has circulated in domestic birds for several months. This hypothesis is strongly supported by a recent study of Munster et al. (*7*), who showed that minor genetic and antigenic diversity exists between H5 and H7 LPAI viruses found in wild birds and those that caused HPAI outbreaks in domestic poultry in Europe. The scientific evidence collected in recent years leads to the conclusion that not only must HPAI viruses be controlled in domestic populations, but LPAI viruses of the H5 and H7 subtypes should also be controlled because they represent HPAI precursors.

## Prevention of Avian Influenza

From December 1999 through April 2003, >50 million birds died or were depopulated after HPAI infection in the European Union (*1*), causing severe economic losses to the private and public sectors. These losses suggest that the strategies and control measures used to combat the disease need improvement, from disease control and animal welfare perspectives.

AI viruses are introduced to domestic poultry primarily through direct or indirect contact with infected birds. Transmission may occur through movement of infected poultry; movement of contaminated equipment, fomites, or vehicles; and exposure to contaminated infectious organic material. Airborne transmission over long distances between farms has not yet been demonstrated. For these reasons, if biosecurity measures are implemented at the farm level, AI infections can be prevented.

Outbreaks that involve large numbers of animals are characterized by the penetration of infection into the commercial circuit; that is, industrially reared poultry and all other poultry that is traded, including those from semi-intensive and backyard farms. Biosecurity (encompassing bioexclusion and biocontainment) represents the first and most important means of prevention. If biosecurity measures of a high standard are implemented and maintained, they create a firewall against infection penetration and perpetuation in the industrial circuit. However, breaches in biosecurity systems do occur. On one hand, the occurrence and extent of the breach should be evaluated and corrective measures should follow; on the other, they indicate the need to establish early warning systems and additional control tools for AI.

## General Aspects of Vaccination

Until recently, AI infections caused by viruses of the H5 and H7 subtype occurred rarely, and vaccination was not considered because stamping out was the recommended control option. Primarily for this reason, vaccinology for AI has not grown at the same rate as for other infectious diseases of animals. Data are being generated from experimental and field research in AI vaccinology, but the rather complex task of vaccinating poultry in different farming and ecologic environments still has areas of uncertainty.

Guidelines on disease prevention and control have been issued as joint recommendations of the World Organization for Animal Health (OIE), the Food and Agriculture Organization (FAO), and the World Health Organization (*8*). These recommendations, however, need to be put into practice in a variety of different field situations; the applicability of 1 system rather than another in a given situation must be evaluated, weighing the benefits of a successful result against the drawbacks of failure.

Vaccination can be a powerful tool to support eradication programs if used in conjunction with other control methods. Vaccination has been shown to increase resistance to field challenge, reduce shedding levels in vaccinated birds, and reduce transmission (*9**,**10*). All these effects of vaccination contribute to controlling AI; however, experience has shown that, to be successful in controlling and ultimately in eradicating the infection, vaccination programs must be part of a wider control strategy that includes biosecurity and monitoring the evolution of infection.

To eradicate AI, the vaccination system must allow the detection of field exposure in a vaccinated flock, which can be achieved by using conventional inactivated vaccines and recombinant vector vaccines. Conventional inactivated vaccines that contain the same viral subtype as the field virus enable detection of field exposure when unvaccinated sentinels left in the flock are tested regularly. This system is applicable in the field but is rather impracticable, especially for the identification of sentinel birds in premises that contain floor-raised birds. A more encouraging system, based on the detection of anti-NS1 antibodies, has been recently developed and can be used with all inactivated vaccines, provided they have the same hemagglutinin subtype as the field virus (*11*). This system is based on the fact that the NS1 protein is synthesized only during active viral replication and, therefore, is rarely present in inactivated vaccines. Birds vaccinated with such vaccines will develop antibodies to NS1 only after field exposure. Full and field testing of this system under different circumstances are still in progress (*11**,**12*), and results should be available before this system is recommended.

To date, the only system that enables detection of field exposure in a vaccinated population and that has resulted in eradication is based on heterologous vaccination and known as "DIVA" (differentiating infected from vaccinated animals). This system was developed to support the eradication programs in the presence of several introductions of LPAI viruses of the H7 subtype (*1**,**9*). Briefly, a vaccine is used that contains a virus possessing the same hemagglutinin, but a different neuraminidase, as the field virus. This vaccination strategy enables detection of antibodies to the neuraminidase antigen of the field virus. For example, a vaccine containing an H7N3 virus can be used against a field virus of the H7N1 subtype. Antibodies to H7 are cross-protective, thus ensuring clinical protection, increased resistance to challenge, and reduction of shedding, while antibodies to the neuraminidase of the field virus (in this case N1) can be used as a natural marker of infection. Experimental data on the quantification of the vaccination effect on transmission within a flock indicate that the reproduction ratio can be reduced to <1 by 1 week after vaccination (*10*). Such a reproduction ratio indicates minor rather than major spread of infection. In simple terms, such vaccination interventions will substantially reduce (although not prevent) secondary outbreaks, depending on the immune status of contact birds and flock.

Promising results have also been obtained with vaccines generated by reverse genetics (*13*). These vaccines are expected to perform like conventional inactivated vaccines; however, data are not yet available as to their efficacy under field conditions. Recombinant fowlpox vaccines that express the hemagglutinin protein of the field virus have also been reported to be efficacious for reducing shedding levels and providing clinical protection (*14*). They enable the detection of field exposure because vaccinated unexposed animals do not have antibodies to any of the other viral proteins. Any test developed to detect antibodies to the nucleoprotein, matrix, NS1, or neuraminidase of the field virus can be used to identify field-exposed birds in a vaccinated population. However, the performance of these vaccines in relation to the immune status of the host to the vector virus is unclear (*15*). Recent encouraging studies indicate that vaccination of day-old chicks with maternal antibodies against fowlpox has been successful. Data are lacking on the performances of such vaccines in a population that has been field exposed to fowlpox. Another aspect that must be carefully considered is the host. These vaccines are likely to induce protective immunity only in birds that are susceptible to infection with the vector virus.

Regardless of the vaccine and companion test used, mapping occurrence of infection within the vaccinated population is imperative, primarily to monitor the evolution of infection and to appropriately manage field-exposed flocks. Field exposure represents a means by which infectious virus may continue to circulate in the immune population; for this reason, vaccination can be considered as only part of a control strategy based on biosecurity, monitoring, approved marketing procedures, and stamping out. An inappropriately managed vaccination campaign will likely result in the virus becoming endemic.

Inadequate biosecurity or vaccination practices can lead to transmission between flocks and selection of variants that exhibit antigenic drift. Antigenic drift of H5N2 viruses belonging to the Mexico lineage, resulting in lower identity (less similarity) to the vaccine strain, has been described (*16*). Extensive use of vaccine in Mexico has resulted in the emergence of antigenic variants that escape the immune response induced by the vaccine. This occurrence is similar to antigenic drift that typically occurs in animals with a long lifespan (pigs and horses) that are routinely vaccinated and in human beings. Mexico has been vaccinating poultry since the HPAI outbreak in 1994 without applying the DIVA principle. Although no HPAI virus has been reported since the implementation of the vaccination campaign, LPAI viruses continue to circulate. Conversely, a similar approach in Pakistan after the HPAI H7N3 outbreaks in 1995 resulted in the isolation of HPAI H7N3 virus ≈10 years later, in 2004 (*17*).

The international scientific community is debating how vaccination of poultry would affect human health. On one hand, vaccinated birds shed less virus; on the other, they do not show any clinical signs of disease and could therefore act as silent carriers. Several factors contribute to the development of infection in humans: insufficient hygienic standards, the characteristics of the strain, and presence of viral dose sufficient to infect a human being. The possibility that vaccinated poultry may not shed enough virus to infect a human being is substantiated by recent field evidence. With reference to the H5N1 crisis, several countries are using vaccination to support control efforts. Vietnam implemented a nationwide vaccination campaign, which was completed in early 2006. The campaign's main achievement is that despite 61 cases of human infection between January and November 2005, no human cases of AI have been reported in Vietnam after December 2005 (*18*).

### Emergency Vaccination

Recent outbreaks in developed countries, notwithstanding their efficient veterinary infrastructures and modern diagnostic systems, have resulted in the culling of millions of birds. Since the year 2000, AI epidemics in areas densely populated with poultry have resulted in 13 million dead birds in Italy in 1999–2000 (H7N1), 5 million dead birds in the United States in 2002 (H7N2), 30 million in the Netherlands in 2003, and 17 million in Canada in 2004. For each of these episodes, biosecurity measures implemented at the farm level were insufficient to prevent massive spread of AI.

Emergency vaccination for AI has become an acceptable tool, in conjunction with other measures, for combating the spread of AI. Using emergency vaccination to reduce the transmission rate could provide an alternative to preemptive culling to reduce the susceptibility of healthy flocks at risk. The effectiveness of such a program depends on variables such as the density of poultry flocks in the area, level of biosecurity and its integration into the industry, characteristics of the virus strain involved, and practical and logistical issues such as vaccine availability and adequate and speedy administration. For this reason, contingency plans that include decision-making patterns under different scenarios should be formulated.

Pivotal work on emergency vaccination has been done in Italy. Application of the DIVA strategy has resulted in the approval of the use of vaccination as an additional tool for the eradication of 2 epidemics of LPAI (H7N1 and H7N3) without massive preemptive killing of animals. Vaccination complemented restriction measures already in place and was integrated into an intensive monitoring program that identified viral circulation in the area (*9*) and culled infected birds. In 2000, heterologous vaccination against an H7 virus was used for the first time in the field as a natural marker vaccine. Subsequently, a DIVA strategy was used by Hong Kong to prevent the introduction of H5N1 into its territories (*19*).

Although use of a DIVA system enabled international trade of poultry products to continue (*9**,**20*), vaccination for AI is a new concept, which several countries are reluctant to even consider. Government authorities ultimately decide whether vaccination should be used in a given country; their reluctance is probably driven by legislative and scientific uncertainties, coupled with doubts about how this practice will be used in the field and other considerations such as exit strategy. With reference to trade implications, a new chapter of the OIE Terrestrial Animal Health Code on AI (*21*) enables the continuation of trade in presence of vaccination if the exporting country is able to produce surveillance and other data that confirm that notifiable avian influenza is not present in the compartment from which the exports come. This chapter is the result of extensive work by OIE experts and the OIE Central Bureau on the issue of reducing the effect of animal diseases through the use of vaccination and is contained in a recommendation document issued as a result of an international conference held in Buenos Aires (April 14–17, 2004) that strongly supports the use of vaccines for diseases on list A (*22*).

### Prophylactic Vaccination

Prophylactic vaccination for viruses of the H5 and H7 subtypes is a completely innovative concept, primarily because only recently have cost-effective situations been identified. Prophylactic vaccination should generate a level of protective immunity in the target population; the immune response may be boosted if a field virus is introduced. Prophylactic vaccination should increase the resistance of birds and, in the case of virus introduction, reduce levels of viral shedding, provided the same levels of biosecurity are maintained. It should be perceived as a tool to maximize biosecurity measures when risk of exposure is high. Ideally, it should prevent the index case. Alternatively, it should reduce the number of secondary outbreaks, thus minimizing the negative effects on animal welfare and potential economic losses in areas where the density of the poultry population would otherwise result in uncontrollable spread without preemptive culling.

Prophylactic vaccination should be considered only when circumstantial evidence indicates that a given area is at risk. Risk for infection may be divided into 2 categories: 1) high risk for infection with either H5 or H7 subtype (e.g., from migratory birds), and 2) risk for infection with a known subtype (e.g., H7N2 in live bird markets in the United States, countries with high exposure to H5N1). For the first category, a bivalent (H5 and H7) vaccination program could be implemented. Italy has recently implemented such a program in the densely populated poultry area at risk for infection (*23*). For the second category, a monovalent (H5 or H7) program would be sufficient.

The choice of vaccine is crucial to the outcome of prophylactic vaccination campaigns. Ideally, vaccines that enable detection of field exposure with any AI virus should be used. Such candidates would be vaccines that enable the identification of field-exposed flocks through the detection of antibodies to an antigen that is common to all type A influenza viruses such as NP, M, or NS1. Such a strategy would detect the introduction of any subtype of AI.

The DIVA system, which uses heterologous neuraminidase, has some limitations in its application for prophylaxis or in situations with risk for introduction of multiple AI subtypes because the system was originally developed to fight a known subtype of AI. The main problem is that the virus against which vaccination is directed must have a different N subtype than the virus present in the vaccine, which, for prophylactic vaccination, is impossible to establish beforehand. An approach to resolving this difficulty is to use seed vaccine strains of the H5 and H7 subtypes that are exhibiting rare neuraminidase subtypes such as N5 or N8. This selection criterion of vaccine strains will greatly reduce the chance that an AI virus of a similar N subtype is introduced. In any case, for surveillance purposes, unvaccinated sentinels should be present in the flock.

Prophylactic vaccination should be continued as long as risk for infection exists. It can be used in a targeted manner for limited periods of time, which requires a detailed exit strategy.

## Conclusions

The scientific veterinary community must control AI infections in poultry for several reasons: to manage the pandemic potential, to preserve profitability of the poultry industry, and to guarantee food security to developing countries. Although biosecurity is recognized as an excellent means of preventing infection, in certain situations the biosecurity standards necessary to prevent infection are difficult to sustain. Vaccination is a potentially powerful tool for supporting eradication programs by increasing the resistance of birds to field challenge and by reducing the amount and duration of virus shed in the environment. Vaccination strategies that encompass monitoring of infection in the field are crucial to the success of such efforts.

Timely information is needed about the efficacy of vaccination in a variety of different avian species, bearing in mind the diverse farming systems used in developed and developing countries. The outcome of such efforts should be made available to the international community because decision makers lack enough information to make educated choices. An enormous effort is required from national governments and funding bodies to make resources available to research programs to develop improved control measures that can be applied under different local conditions. To maximize the global effort to combat this disease, developing and sustaining transversal research programs on AI control, which encompass veterinary and agricultural science, are imperative.
